# Tetra­aqua­bis­(2-{[5-(pyridin-4-yl)-1,3,4-oxadiazol-2-yl]sulfan­yl}acetato)­cobalt(II) monohydrate

**DOI:** 10.1107/S1600536811046526

**Published:** 2011-11-12

**Authors:** Guang-Rui Yang, Guo-Ting Li

**Affiliations:** a Department of Environmental and Municipal Engineering, North China University of Water Conservancy and Electric Power, Zhengzhou 450011, People’s Republic of China

## Abstract

In the title compound, [Co(C_9_H_6_N_3_O_3_S)_2_(H_2_O)_4_]·H_2_O, the two 2-{[5-(pyridin-4-yl)-1,3,4-oxadiazol-2-yl]sulfan­yl}acetate ligands are monodentate. One coordinates the metal atom *via* the pyridyl N atom whereas the other coordinates *via* the carboxyl­ate O atom. The Co^II^ atom adopts a slightly distorted octa­hedral coordination geometry with four O atoms of the coordinated water mol­ecules located in the equatorial plane and the N and O atoms of the two POA ligands in axial positions. In the crystal, the components are connected through O—H⋯O and O—H⋯N hydrogen bonds into a three-dimensional framework.

## Related literature

For metal-assisted transformation of *N*-benzoyl­dithio­carbazate to 5-phenyl-1,3,4-oxadiazole-2-thiol (pot) in the presence of ethyl­enediamine, and its transition metal complexes, see: Tripathi *et al.* (2007 ▶). For zinc and cadmium metal-organic polymers formed with 5-(4-pyrid­yl)-1,3,4-oxadiazole-2-thiol, see: Du *et al.* (2006 ▶). For the synthesis of 5-(4-pyrid­yl)-1,3,4-oxadiazole-2-thiol, see: Young & Wood (1955 ▶).
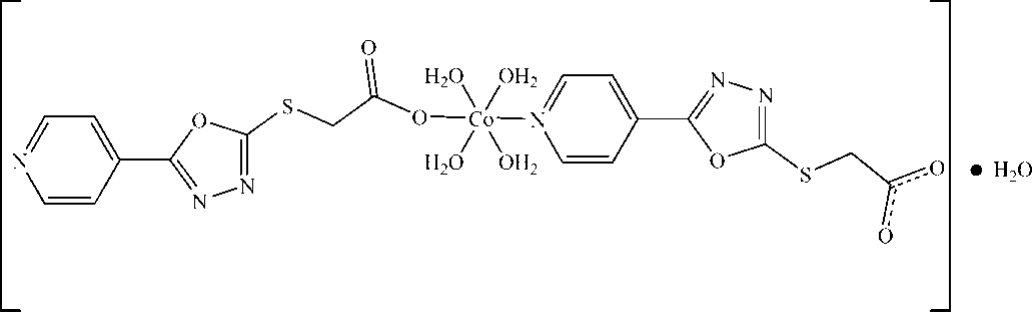


## Experimental

### 

#### Crystal data

[Co(C_9_H_6_N_3_O_3_S)_2_(H_2_O)_4_]·H_2_O
*M* *_r_* = 621.47
Triclinic, 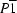

*a* = 7.393 (4) Å
*b* = 11.122 (6) Å
*c* = 16.014 (8) Å
α = 103.904 (6)°
β = 96.040 (6)°
γ = 103.017 (6)°
*V* = 1227.5 (11) Å^3^
*Z* = 2
Mo *K*α radiation
μ = 0.94 mm^−1^
*T* = 293 K
0.40 × 0.25 × 0.15 mm

#### Data collection

Siemens SMART CCD diffractometer
8804 measured reflections
4234 independent reflections
2975 reflections with *I* > 2σ(*I*)
*R*_int_ = 0.056

#### Refinement

*R*[*F* ^2^ > 2σ(*F* ^2^)] = 0.065
*wR*(*F* ^2^) = 0.182
*S* = 1.00
4234 reflections
373 parameters
14 restraints
H atoms treated by a mixture of independent and constrained refinement
Δρ_max_ = 1.51 e Å^−3^
Δρ_min_ = −0.82 e Å^−3^

### 

Data collection: *SMART* (Siemens, 1996 ▶); cell refinement: *SAINT* (Siemens, 1994 ▶); data reduction: *SAINT*; program(s) used to solve structure: *SHELXS97* (Sheldrick, 2008 ▶); program(s) used to refine structure: *SHELXL97* (Sheldrick, 2008 ▶); molecular graphics: *SHELXTL* (Sheldrick, 2008 ▶); software used to prepare material for publication: *SHELXL97*.

## Supplementary Material

Crystal structure: contains datablock(s) I, global. DOI: 10.1107/S1600536811046526/gk2415sup1.cif

Structure factors: contains datablock(s) I. DOI: 10.1107/S1600536811046526/gk2415Isup2.hkl

Additional supplementary materials:  crystallographic information; 3D view; checkCIF report

## Figures and Tables

**Table 1:** Selected bond lengths (Å)

Co1—O8	2.074 (4)
Co1—O9	2.080 (4)
Co1—O10	2.083 (4)
Co1—O1	2.107 (3)
Co1—O7	2.135 (4)
Co1—N4	2.164 (4)

**Table 2:** Hydrogen-bond geometry (Å, °)

*D*—H⋯*A*	*D*—H	H⋯*A*	*D*⋯*A*	*D*—H⋯*A*
O10—H10*B*⋯O2	0.84 (1)	1.77 (1)	2.608 (5)	172 (6)
O11—H11*D*⋯O7	0.88 (1)	2.04 (1)	2.886 (5)	163 (2)
O10—H10*A*⋯O11^i^	0.84 (1)	1.97 (1)	2.810 (6)	178 (6)
O7—H7*A*⋯O5^ii^	0.84 (1)	1.94 (2)	2.741 (5)	160 (5)
O8—H8*A*⋯O4^ii^	0.84 (1)	1.93 (2)	2.767 (5)	172 (6)
O9—H9*B*⋯O11^iii^	0.84 (1)	1.96 (1)	2.793 (5)	174 (6)
O7—H7*B*⋯O4^iv^	0.84 (1)	1.97 (3)	2.762 (5)	156 (6)
O8—H8*B*⋯O1^v^	0.84 (1)	1.87 (2)	2.677 (5)	163 (6)
O9—H9*A*⋯O5^vi^	0.84 (1)	1.93 (2)	2.761 (5)	170 (5)
O11—H11*C*⋯N3^vii^	0.83 (1)	1.98 (2)	2.785 (6)	165 (5)

Symmetry codes: (i) 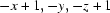; (ii) 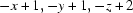; (iii) 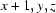; (iv) 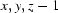; (v) 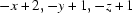; (vi) 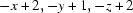; (vii) 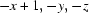.

**Fig. 1.**
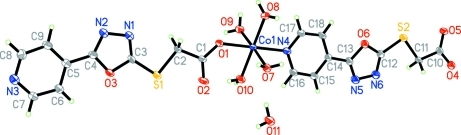
ORTEP diagram of of the title compound with 30% probability ellipsoids for all non-hydrogen atoms.

**Fig. 2.**
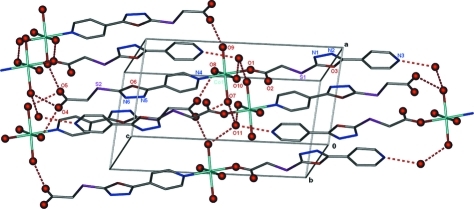
A complicated hydrogen-bond network in the title compound (1).

## supplementary crystallographic information

### Comment

Recently, pyridyl-containing 1,3,4-oxadiazole-2-thiones have been systematically explored as promising bridging ligands in coordination chemistry (Du *et al.*, 2006; Tripathi *et al.*, 2007). Our contribution to these studies is synthesis of the multifunctional ligand ,{[5-(pyridin-4-yl)-1,3,4-oxadiazol-2-yl]sulfanyl}acetic acid (HPOA), and reported herein structure of a new complex [Co(POA)_2_(H_2_O)_4_].H_2_O (I).

As shown in Fig. 1 (I) is a mononuclear complex. The asymmetric unit consists of the complex molecule and one water of crystallization. In (I) the Co^II^ center is ligated by four O atoms from four water molecules located in the equatorial plane, and two monodentate POA anions. One POA anion coordinates the metal center *via* the pyridyl N atom whereas the other *via* the carboxylate O atom. The Co^II^ ion is in a slightly distorted octahedral coordination environement with the in-plane and axial-trans angles being 175.09 (15), 177.66 (14) and 178.54 (13)°, and the bond distances Co—O and Co—N ranging from 2.074 (4) to 2.164 (4) Å.

In (I) the hydrogen-bonding interactions result in a 3D supramolecular network as shown in Fig. 2. The 3D hydrogen-bonded network is stabilized through the intermolecular π···π interactions with a center-to-center distance of pyridyl groups being 3.662 Å and a center-to-center distance of oxadiazole groups being 3.375 Å, respectively. There are complicated hydrogen-bonding system in (I): each coordination water molecule forms two O—H···O hydrogen bonds while every uncoordinated carboxyl group of POA in one monomer adopts bridging and chelating coordination modes to links with two other monomers through the formed three O—H···O hydrogen bonds, and especially the lattice water O11 acting as a tetrahedral hydrogen-bonding connector binds with four monomers (1) through three O···O hydrogen bonds and one O···N hydrogen bond. In this way monomers of (I) are linked into the 3D supramolecular architecture.

### Experimental

The sodium salt of 2-(5-(pyridin-4-yl)-1,3,4-oxadiazol-2-ylthio)acetic acid (HPOA) was synthesized in the following process. To a solution of sodium hydroxide (1.60 g, 40 mmol) and 95% alcohol (50 mL) was added 5-pyridyl-2-mercapto-1,3,4-oxadiazole (3.58 g, 20 mmol) and the resulting mixture was refluxed for half an hour. And then a solution of chloroactic acid (1.89 g, 20 mmol) and 95% alcohol (70 mL) was dropwise added to the mixture with continuous refluxing for 3 hours. Pale yellow precipitate was filtered. After recrystallized from alcohol/water (2:1), the obtained pure product was 2.76 g. Yield: 51%. Selected IR (cm^-1^, KBr pellet): 3489(w), 1598(*s*),

1464(*m*), 1402(*s*), 1220(*m*), 1190(*m*), 1084(*m*), 909(*m*), 835(*m*), 704(w), 519(*m*).

The title compound (1), was prepared according to the following process. A mixture of NaPOA (51.8 mg, 0.2 mmol), CoCl_2_.6H_2_O (23.8 mg, 0.1 mmol) and deionized water (20 ml) was stirred for 30 minutes and then filtered. The filtrate was allowed to evaporate at room temperature for a week, and then red block crystals were obtain in 57% yield. Selected IR (cm^-1^, KBr pellet): 3228(*m*), 1571(*s*), 1496(*m*), 1449(*s*), 142(*m*), 1390(*s*), 1226(*s*), 1197(*m*), 1062(*m*), 1004(*s*), 873(w), 841(*m*), 800(w), 710(*s*), 584(w), 523(w).

### Refinement

The H atoms of water molecules were located from difference Fourier maps and refined with restraints imposed on O-H and H···H distances and with U_iso_(H) = 1.5U_eq_(O). The remaining hydrogen atom positions were generated geometrically. All H atoms were allowed to ride on their parent atoms with U_iso_(H) = 1.5U_eq_(C).

### Crystal data

**Table:**

[Co(C_9_H_6_N_3_O_3_S)_2_(H_2_O)_4_]·H_2_O	*Z* = 2
*M**_r_* = 621.47	*F*(000) = 638
Triclinic, *P*1	*D*_x_ = 1.681 Mg m^−^^3^
Hall symbol: -P 1	Mo *K*α radiation, λ = 0.71073 Å
*a* = 7.393 (4) Å	Cell parameters from 2275 reflections
*b* = 11.122 (6) Å	θ = 2.7–25.1°
*c* = 16.014 (8) Å	µ = 0.94 mm^−^^1^
α = 103.904 (6)°	*T* = 293 K
β = 96.040 (6)°	Block, red
γ = 103.017 (6)°	0.40 × 0.25 × 0.15 mm
*V* = 1227.5 (11) Å^3^	

### Data collection

**Table:**

Siemens SMART CCD diffractometer	2975 reflections with *I* > 2σ(*I*)
Radiation source: fine-focus sealed tube	*R*_int_ = 0.056
graphite	θ_max_ = 25.0°, θ_min_ = 2.7°
ω scan	*h* = −8→8
8804 measured reflections	*k* = −13→13
4234 independent reflections	*l* = −19→18

### Refinement

**Table:**

Refinement on *F*^2^	Primary atom site location: structure-invariant direct methods
Least-squares matrix: full	Secondary atom site location: difference Fourier map
*R*[*F*^2^ > 2σ(*F*^2^)] = 0.065	Hydrogen site location: inferred from neighbouring sites
*wR*(*F*^2^) = 0.182	H atoms treated by a mixture of independent and constrained refinement
*S* = 1.00	*w* = 1/[σ^2^(*F*_o_^2^) + (0.1108*P*)^2^] where *P* = (*F*_o_^2^ + 2*F*_c_^2^)/3
4234 reflections	(Δ/σ)_max_ < 0.001
373 parameters	Δρ_max_ = 1.51 e Å^−^^3^
14 restraints	Δρ_min_ = −0.82 e Å^−^^3^

### Special details

**Table:**

Geometry. All esds (except the esd in the dihedral angle between two l.s. planes) are estimated using the full covariance matrix. The cell esds are taken into account individually in the estimation of esds in distances, angles and torsion angles; correlations between esds in cell parameters are only used when they are defined by crystal symmetry. An approximate (isotropic) treatment of cell esds is used for estimating esds involving l.s. planes.
Refinement. Refinement of F^2^ against ALL reflections. The weighted R-factor wR and goodness of fit S are based on F^2^, conventional R-factors R are based on F, with F set to zero for negative F^2^. The threshold expression of F^2^ > 2sigma(F^2^) is used only for calculating R-factors(gt) etc. and is not relevant to the choice of reflections for refinement. R-factors based on F^2^ are statistically about twice as large as those based on F, and R- factors based on ALL data will be even larger.

### Fractional atomic coordinates and isotropic or equivalent isotropic displacement parameters (Å^2^)

**Table:**

	*x*	*y*	*z*	*U*_iso_*/*U*_eq_	
Co1	0.78325 (9)	0.30500 (6)	0.54979 (4)	0.0341 (2)	
S1	0.75640 (19)	0.10713 (12)	0.18118 (8)	0.0428 (4)	
S2	0.63708 (19)	0.46656 (12)	1.16857 (8)	0.0440 (4)	
O1	0.8385 (5)	0.3050 (3)	0.4234 (2)	0.0402 (8)	
O2	0.6631 (6)	0.1130 (3)	0.3422 (2)	0.0572 (10)	
O3	0.8313 (5)	0.0864 (3)	0.0228 (2)	0.0405 (8)	
O4	0.3915 (5)	0.3743 (3)	1.3574 (2)	0.0462 (9)	
O5	0.6136 (5)	0.5403 (3)	1.3476 (2)	0.0497 (9)	
O6	0.6559 (5)	0.4033 (3)	0.9999 (2)	0.0424 (8)	
O7	0.5077 (5)	0.3243 (3)	0.5123 (2)	0.0389 (8)	
O8	0.8788 (5)	0.5034 (3)	0.5868 (2)	0.0457 (9)	
O9	1.0481 (5)	0.2784 (4)	0.5833 (3)	0.0483 (9)	
O10	0.6705 (5)	0.1073 (3)	0.5044 (2)	0.0452 (9)	
O11	0.1925 (6)	0.1010 (4)	0.4736 (2)	0.0570 (10)	
N1	0.9855 (6)	0.2754 (4)	0.1118 (3)	0.0473 (11)	
N2	1.0274 (6)	0.2712 (4)	0.0278 (3)	0.0481 (11)	
N3	0.8966 (6)	0.0086 (4)	−0.2939 (3)	0.0492 (11)	
N4	0.7224 (5)	0.3085 (4)	0.6795 (3)	0.0362 (9)	
N5	0.4802 (7)	0.2040 (4)	0.9494 (3)	0.0505 (12)	
N6	0.4768 (6)	0.2448 (4)	1.0384 (3)	0.0487 (11)	
C1	0.7812 (6)	0.2151 (4)	0.3525 (3)	0.0350 (11)	
C2	0.8695 (7)	0.2403 (5)	0.2745 (3)	0.0399 (12)	
H2A	1.0065	0.2479	0.2856	0.048*	
H2B	0.8508	0.3211	0.2641	0.048*	
C3	0.8697 (7)	0.1655 (5)	0.1053 (3)	0.0363 (11)	
C4	0.9354 (7)	0.1611 (4)	−0.0218 (3)	0.0370 (11)	
C5	0.9225 (7)	0.1068 (5)	−0.1155 (3)	0.0379 (11)	
C6	0.7994 (7)	−0.0095 (5)	−0.1589 (3)	0.0436 (12)	
H6	0.7221	−0.0581	−0.1285	0.052*	
C7	0.7907 (7)	−0.0541 (5)	−0.2484 (3)	0.0445 (12)	
H7	0.7041	−0.1339	−0.2785	0.053*	
C8	1.0126 (8)	0.1215 (5)	−0.2516 (3)	0.0477 (13)	
H8	1.0851	0.1686	−0.2845	0.057*	
C9	1.0345 (7)	0.1750 (5)	−0.1624 (3)	0.0425 (12)	
H9	1.1224	0.2551	−0.1343	0.051*	
C10	0.4996 (7)	0.4344 (5)	1.3167 (3)	0.0376 (11)	
C11	0.4767 (7)	0.3688 (5)	1.2190 (3)	0.0377 (11)	
H11A	0.3455	0.3558	1.1908	0.045*	
H11B	0.5032	0.2837	1.2108	0.045*	
C12	0.5816 (7)	0.3609 (5)	1.0651 (3)	0.0381 (11)	
C13	0.5860 (7)	0.2988 (5)	0.9296 (3)	0.0391 (12)	
C14	0.6358 (6)	0.3042 (4)	0.8441 (3)	0.0332 (10)	
C15	0.5456 (7)	0.2050 (5)	0.7710 (3)	0.0412 (12)	
H15	0.4529	0.1337	0.7760	0.049*	
C16	0.5918 (7)	0.2109 (5)	0.6909 (3)	0.0405 (12)	
H16	0.5283	0.1426	0.6412	0.049*	
C17	0.8101 (7)	0.4028 (5)	0.7512 (3)	0.0398 (12)	
H17	0.9044	0.4719	0.7446	0.048*	
C18	0.7718 (7)	0.4056 (5)	0.8338 (3)	0.0408 (12)	
H18	0.8369	0.4753	0.8826	0.049*	
H7B	0.503 (8)	0.355 (5)	0.469 (2)	0.061*	
H7A	0.471 (8)	0.379 (4)	0.548 (3)	0.061*	
H8A	0.793 (6)	0.540 (5)	0.599 (4)	0.061*	
H9B	1.087 (8)	0.226 (4)	0.547 (3)	0.061*	
H9A	1.144 (5)	0.340 (3)	0.604 (4)	0.061*	
H8B	0.966 (5)	0.554 (4)	0.573 (4)	0.061*	
H10A	0.709 (8)	0.044 (4)	0.512 (4)	0.061*	
H11C	0.163 (6)	0.082 (5)	0.4196 (8)	0.061*	
H10B	0.657 (8)	0.105 (6)	0.4512 (12)	0.061*	
H11D	0.3009 (12)	0.1571 (12)	0.481 (3)	0.061*	

### Atomic displacement parameters (Å^2^)

**Table:**

	*U*^11^	*U*^22^	*U*^33^	*U*^12^	*U*^13^	*U*^23^
Co1	0.0346 (4)	0.0291 (4)	0.0268 (4)	−0.0062 (3)	0.0043 (3)	−0.0008 (3)
S1	0.0500 (8)	0.0355 (7)	0.0281 (6)	−0.0053 (6)	0.0019 (6)	−0.0019 (5)
S2	0.0508 (8)	0.0391 (7)	0.0300 (7)	−0.0028 (6)	0.0082 (6)	−0.0001 (5)
O1	0.044 (2)	0.0313 (17)	0.0313 (18)	−0.0080 (16)	0.0090 (15)	−0.0019 (14)
O2	0.064 (2)	0.044 (2)	0.035 (2)	−0.025 (2)	0.0006 (18)	−0.0034 (16)
O3	0.049 (2)	0.0304 (18)	0.0276 (17)	−0.0054 (16)	0.0009 (15)	−0.0017 (14)
O4	0.057 (2)	0.043 (2)	0.0319 (18)	0.0023 (18)	0.0133 (17)	0.0046 (16)
O5	0.045 (2)	0.046 (2)	0.0345 (19)	−0.0126 (18)	0.0057 (16)	−0.0090 (16)
O6	0.052 (2)	0.0395 (19)	0.0273 (18)	0.0007 (17)	0.0102 (16)	0.0036 (15)
O7	0.040 (2)	0.039 (2)	0.0289 (18)	0.0010 (16)	0.0042 (15)	0.0029 (15)
O8	0.045 (2)	0.0286 (19)	0.052 (2)	−0.0057 (16)	0.0163 (19)	0.0014 (17)
O9	0.036 (2)	0.045 (2)	0.048 (2)	−0.0013 (17)	0.0039 (17)	−0.0032 (18)
O10	0.058 (2)	0.0292 (18)	0.038 (2)	−0.0055 (17)	0.0087 (18)	0.0054 (17)
O11	0.062 (2)	0.051 (2)	0.040 (2)	−0.005 (2)	0.0034 (19)	−0.0016 (18)
N1	0.051 (3)	0.041 (2)	0.035 (2)	−0.004 (2)	0.008 (2)	−0.0046 (19)
N2	0.053 (3)	0.042 (3)	0.033 (2)	−0.005 (2)	0.007 (2)	−0.002 (2)
N3	0.054 (3)	0.049 (3)	0.035 (2)	0.002 (2)	0.003 (2)	0.005 (2)
N4	0.036 (2)	0.031 (2)	0.033 (2)	−0.0024 (18)	0.0025 (17)	0.0020 (17)
N5	0.060 (3)	0.048 (3)	0.031 (2)	−0.005 (2)	0.010 (2)	0.003 (2)
N6	0.056 (3)	0.049 (3)	0.031 (2)	−0.002 (2)	0.013 (2)	0.006 (2)
C1	0.033 (3)	0.031 (3)	0.029 (2)	−0.004 (2)	−0.001 (2)	0.000 (2)
C2	0.046 (3)	0.034 (3)	0.027 (2)	−0.004 (2)	0.006 (2)	−0.002 (2)
C3	0.038 (3)	0.036 (3)	0.024 (2)	0.004 (2)	0.000 (2)	−0.005 (2)
C4	0.035 (3)	0.030 (3)	0.039 (3)	0.003 (2)	0.003 (2)	0.002 (2)
C5	0.040 (3)	0.033 (3)	0.035 (3)	0.008 (2)	0.001 (2)	0.004 (2)
C6	0.048 (3)	0.040 (3)	0.033 (3)	0.000 (3)	0.004 (2)	0.004 (2)
C7	0.040 (3)	0.043 (3)	0.038 (3)	0.000 (2)	0.001 (2)	0.001 (2)
C8	0.051 (3)	0.053 (3)	0.036 (3)	0.008 (3)	0.007 (2)	0.012 (3)
C9	0.044 (3)	0.033 (3)	0.041 (3)	0.001 (2)	−0.001 (2)	0.003 (2)
C10	0.042 (3)	0.040 (3)	0.027 (2)	0.011 (2)	0.004 (2)	0.002 (2)
C11	0.039 (3)	0.034 (3)	0.029 (2)	−0.001 (2)	0.003 (2)	−0.001 (2)
C12	0.040 (3)	0.039 (3)	0.029 (3)	0.006 (2)	0.003 (2)	0.003 (2)
C13	0.046 (3)	0.036 (3)	0.028 (3)	0.005 (2)	0.006 (2)	0.000 (2)
C14	0.036 (3)	0.032 (2)	0.030 (2)	0.005 (2)	0.008 (2)	0.006 (2)
C15	0.045 (3)	0.035 (3)	0.033 (3)	−0.006 (2)	0.008 (2)	0.004 (2)
C16	0.046 (3)	0.034 (3)	0.028 (2)	−0.005 (2)	0.003 (2)	−0.001 (2)
C17	0.041 (3)	0.035 (3)	0.032 (3)	−0.007 (2)	0.006 (2)	0.003 (2)
C18	0.044 (3)	0.035 (3)	0.028 (2)	−0.006 (2)	−0.001 (2)	−0.004 (2)

### Geometric parameters (Å, °)

**Table:**

Co1—O8	2.074 (4)	N3—C7	1.322 (7)
Co1—O9	2.080 (4)	N3—C8	1.325 (7)
Co1—O10	2.083 (4)	N4—C17	1.340 (6)
Co1—O1	2.107 (3)	N4—C16	1.343 (6)
Co1—O7	2.135 (4)	N5—C13	1.291 (6)
Co1—N4	2.164 (4)	N5—N6	1.394 (6)
S1—C3	1.718 (5)	N6—C12	1.290 (7)
S1—C2	1.800 (5)	C1—C2	1.525 (6)
S2—C12	1.732 (5)	C2—H2A	0.9900
S2—C11	1.812 (5)	C2—H2B	0.9900
O1—C1	1.277 (5)	C4—C5	1.461 (7)
O2—C1	1.229 (6)	C5—C6	1.375 (7)
O3—C3	1.359 (5)	C5—C9	1.396 (7)
O3—C4	1.381 (6)	C6—C7	1.389 (7)
O4—C10	1.261 (6)	C6—H6	0.9500
O5—C10	1.236 (6)	C7—H7	0.9500
O6—C12	1.361 (6)	C8—C9	1.386 (7)
O6—C13	1.365 (6)	C8—H8	0.9500
O7—H7B	0.841 (10)	C9—H9	0.9500
O7—H7A	0.843 (10)	C10—C11	1.532 (6)
O8—H8A	0.839 (10)	C11—H11A	0.9900
O8—H8B	0.837 (10)	C11—H11B	0.9900
O9—H9B	0.840 (10)	C13—C14	1.466 (6)
O9—H9A	0.838 (11)	C14—C15	1.385 (7)
O10—H10A	0.842 (7)	C14—C18	1.388 (7)
O10—H10B	0.841 (10)	C15—C16	1.373 (7)
O11—H11C	0.831 (10)	C15—H15	0.9500
O11—H11D	0.875 (8)	C16—H16	0.9500
N1—C3	1.298 (6)	C17—C18	1.377 (6)
N1—N2	1.404 (6)	C17—H17	0.9500
N2—C4	1.280 (6)	C18—H18	0.9500
			
O8—Co1—O9	93.76 (15)	N1—C3—S1	131.2 (4)
O8—Co1—O10	175.09 (15)	O3—C3—S1	116.2 (3)
O9—Co1—O10	90.39 (15)	N2—C4—O3	112.4 (4)
O8—Co1—O1	88.89 (13)	N2—C4—C5	130.1 (5)
O9—Co1—O1	90.15 (14)	O3—C4—C5	117.5 (4)
O10—Co1—O1	88.47 (13)	C6—C5—C9	119.1 (5)
O8—Co1—O7	88.48 (14)	C6—C5—C4	121.3 (5)
O9—Co1—O7	177.66 (14)	C9—C5—C4	119.6 (5)
O10—Co1—O7	87.35 (15)	C5—C6—C7	118.3 (5)
O1—Co1—O7	89.22 (13)	C5—C6—H6	120.8
O8—Co1—N4	90.21 (14)	C7—C6—H6	120.8
O9—Co1—N4	91.06 (15)	N3—C7—C6	123.4 (5)
O10—Co1—N4	92.35 (14)	N3—C7—H7	118.3
O1—Co1—N4	178.54 (13)	C6—C7—H7	118.3
O7—Co1—N4	89.61 (14)	N3—C8—C9	123.9 (5)
C3—S1—C2	97.1 (2)	N3—C8—H8	118.1
C12—S2—C11	96.7 (2)	C9—C8—H8	118.1
C1—O1—Co1	128.8 (3)	C8—C9—C5	117.4 (5)
C3—O3—C4	102.2 (4)	C8—C9—H9	121.3
C12—O6—C13	102.0 (4)	C5—C9—H9	121.3
Co1—O7—H7B	111 (4)	O5—C10—O4	126.2 (5)
Co1—O7—H7A	116 (4)	O5—C10—C11	118.9 (4)
H7B—O7—H7A	99 (5)	O4—C10—C11	114.8 (4)
Co1—O8—H8A	113 (4)	C10—C11—S2	110.1 (3)
Co1—O8—H8B	132 (4)	C10—C11—H11A	109.6
H8A—O8—H8B	109 (5)	S2—C11—H11A	109.6
Co1—O9—H9B	119 (4)	C10—C11—H11B	109.6
Co1—O9—H9A	122 (4)	S2—C11—H11B	109.6
H9B—O9—H9A	103 (6)	H11A—C11—H11B	108.2
Co1—O10—H10A	133 (4)	N6—C12—O6	112.9 (4)
Co1—O10—H10B	95 (4)	N6—C12—S2	129.8 (4)
H10A—O10—H10B	110 (6)	O6—C12—S2	117.4 (4)
H11C—O11—H11D	100.1 (15)	N5—C13—O6	112.5 (4)
C3—N1—N2	106.1 (4)	N5—C13—C14	127.9 (4)
C4—N2—N1	106.7 (4)	O6—C13—C14	119.6 (4)
C7—N3—C8	117.7 (5)	C15—C14—C18	118.5 (4)
C17—N4—C16	116.7 (4)	C15—C14—C13	119.4 (4)
C17—N4—Co1	123.8 (3)	C18—C14—C13	122.1 (4)
C16—N4—Co1	119.5 (3)	C16—C15—C14	119.2 (4)
C13—N5—N6	106.5 (4)	C16—C15—H15	120.4
C12—N6—N5	106.1 (4)	C14—C15—H15	120.4
O2—C1—O1	126.2 (4)	N4—C16—C15	123.3 (4)
O2—C1—C2	118.4 (4)	N4—C16—H16	118.4
O1—C1—C2	115.3 (4)	C15—C16—H16	118.4
C1—C2—S1	107.5 (3)	N4—C17—C18	124.1 (4)
C1—C2—H2A	110.2	N4—C17—H17	118.0
S1—C2—H2A	110.2	C18—C17—H17	118.0
C1—C2—H2B	110.2	C17—C18—C14	118.3 (4)
S1—C2—H2B	110.2	C17—C18—H18	120.9
H2A—C2—H2B	108.5	C14—C18—H18	120.9
N1—C3—O3	112.6 (4)		

### Hydrogen-bond geometry (Å, °)

**Table:**

*D*—H···*A*	*D*—H	H···*A*	*D*···*A*	*D*—H···*A*
O10—H10B···O2	0.84 (1)	1.77 (1)	2.608 (5)	172 (6)
O11—H11D···O7	0.88 (1)	2.04 (1)	2.886 (5)	163 (2)
O10—H10A···O11^i^	0.84 (1)	1.97 (1)	2.810 (6)	178 (6)
O7—H7A···O5^ii^	0.84 (1)	1.94 (2)	2.741 (5)	160 (5)
O8—H8A···O4^ii^	0.84 (1)	1.93 (2)	2.767 (5)	172 (6)
O9—H9B···O11^iii^	0.84 (1)	1.96 (1)	2.793 (5)	174 (6)
O7—H7B···O4^iv^	0.84 (1)	1.97 (3)	2.762 (5)	156 (6)
O8—H8B···O1^v^	0.84 (1)	1.87 (2)	2.677 (5)	163 (6)
O9—H9A···O5^vi^	0.84 (1)	1.93 (2)	2.761 (5)	170 (5)
O11—H11C···N3^vii^	0.83 (1)	1.98 (2)	2.785 (6)	165 (5)

Symmetry codes: (i) −*x*+1, −*y*, −*z*+1; (ii) −*x*+1, −*y*+1, −*z*+2; (iii) *x*+1, *y*, *z*; (iv) *x*, *y*, *z*−1; (v) −*x*+2, −*y*+1, −*z*+1; (vi) −*x*+2, −*y*+1, −*z*+2; (vii) −*x*+1, −*y*, −*z*.
